# Using transcription of six *Puccinia triticina* races to identify the effective secretome during infection of wheat

**DOI:** 10.3389/fpls.2013.00520

**Published:** 2014-01-13

**Authors:** Myron Bruce, Kerri A. Neugebauer, David L. Joly, Pierre Migeon, Christina A. Cuomo, Shichen Wang, Eduard Akhunov, Guus Bakkeren, James A. Kolmer, John P. Fellers

**Affiliations:** ^1^USDA-ARS Hard Winter Wheat Genetics Research Unit, Department of Plant PathologyManhattan, KS, USA; ^2^Department of Plant Pathology, Kansas State UniversityManhattan, KS, USA; ^3^Département de biologie, Université de MonctonMoncton, NB, Canada; ^4^Broad Institute of MIT and HarvardCambridge, MA, USA; ^5^Pacific Agri-Food Research Centre, Agriculture and Agri-Food CanadaSummerland, BC, Canada; ^6^USDA–ARS Cereal Disease Laboratory, Department of Plant Pathology, University of MinnesotaSt. Paul, MN, USA

**Keywords:** *Puccinia triticina*, secreted peptides, effectors, leaf rust, RNA sequencing

## Abstract

Wheat leaf rust, caused by the basidiomycete *Puccinia triticina*, can cause yield losses of up to 20% in wheat producing regions. During infection, the fungus forms haustoria that secrete proteins into the plant cell and effect changes in plant transcription, metabolism, and defense. It is hypothesized that new races emerge as a result of overcoming plant resistance via changes in the secreted effector proteins. To understand gene expression during infection and find genetic differences associated with races, RNA from wheat leaves infected with six different rust races, at 6 days post inoculation, was sequenced using Illumina. As *P. triticina* is an obligate biotroph, RNA from both the host and fungi were present and separated by alignment to the *P. triticina* genome and a wheat EST reference. A total of 222,571 rust contigs were assembled from 165 million reads. An examination of the resulting contigs revealed 532 predicted secreted proteins among the transcripts. Of these, 456 were found in all races. Fifteen genes were found with amino acid changes, corresponding to putative avirulence effectors potentially recognized by 11 different leaf rust resistance (*Lr*) genes. Twelve of the potential avirulence effectors have no homology to known genes. One gene had significant similarity to cerato-platanin, a known fungal elicitor, and another showed similarity to fungal tyrosinase, an enzyme involved in melanin synthesis. Temporal expression profiles were developed for these genes by qRT-PCR and show that the genes expression patterns were consistent between races from infection initiation to just prior to spore eruption.

## Introduction

Wheat leaf rust can cause extensive economic impact on the wheat producing areas of the world, with significant yield losses reported during epidemics. The causative agent of wheat leaf rust is *Puccinia triticina* (*Pt*) which is an obligate biotrophic basidiomycete (Bolton et al., 2008). In the Great Plains of the US, the pathogen spreads by asexual urediniospores that land on the leaf surface and germinate under appropriate conditions. Using thigmotrophic interactions with the surface of the leaf, the germ tube orients itself perpendicular to the leaf veins and grows until it reaches a stomata. The germ tube generates an appressorium over the stomata and penetrates the interior of the leaf. Directly beneath the site of penetration, the haustorial mother cell is formed and infection structures penetrate the plant cell wall to form feeding structures called haustoria. While the haustorium penetrates the plant cell wall, it does not rupture the plant cell membrane. Communication between the growing fungus and the plant occurs across the haustorial membrane-plasma membrane interface in the form of proteins and other small molecules secreted by the fungus. The secreted proteins, some of which may be effectors, perform a variety of functions including host transcriptional reprogramming to benefit pathogen growth and mitigation of host defenses (Bolton et al., 2008).

Plants have several systems of defense to guard against infection of pathogens. Effector triggered immunity is the most distinct and stems from the host's ability to recognize the presence or activity of pathogen effectors. The host cell responds by inducing a localized response to isolate the infection. Originally, this was described at the genetic level by Flor as the “gene for gene” hypothesis (Flor, 1942). Flor's model posits that if a pathogen protein (coded by an avirulence gene) is recognized by a plant protein (coded by a resistance gene, or *R* gene), then resistance is triggered in the plant. This is usually characterized by hypersensitive cell death, or HR (Mur et al., 2008). While the genetics of avirulence in the flax rust (*Melampsora lini*)—flax (*Linum usitatissimum*) pathosystem had been described by Flor (1971), the molecular mechanism underlying the interaction between pathogen avirulence gene and host resistance gene has only become more clear as technologies advance. Dodds et al. validated the theory in flax rust by showing that a member of the gene family, *AvrL567*, is expressed in haustoria and secreted into the plant cell cytoplasm where its activity is recognized (Dodds et al., 2004).

As research has progressed, the activities of various “avirulence” genes have been shown to have a beneficial effect on the pathogen's fitness and/or ability to cause disease. Thus, the corresponding gene products were renamed “effectors.” Effectors from plant pathogenic fungi include protease inhibitors, chitin binding proteins, metalloproteases, and many genes of unknown function (Stergiopoulos and de Wit, 2009). Several effectors from rust pathogens have been described. Rust transferred proteins (RTP) have a demonstrated protease inhibitor function (Pretsch et al., 2013) and RTP1 was recently shown to be a structural effector involved in filament formation in the extra-haustorial matrix (Kemen et al., 2013). RTPs were first described in *Uromyces fabae* (bean rust pathogen) and shown to translocate from the haustorium to the plant cytoplasm (Kemen et al., 2005). *Pt* encodes three proteins from this family, though their function have not been determined (Pretsch et al., 2013).

There are many difficulties encountered with *Pt* as a study system. The fungus is an obligate biotroph which cannot be cultured outside of its host. Additionally, the alternate host, *Thalictrum speciosissium*, is required for the sexual stage of the organism but is not known to be widely present in North America (Bolton et al., 2008). While controlled crosses have been useful in examining heritability of avirulence factors (Samborski and Dyck, 1968; Statler, 2000), the crossing and purification process is time-consuming and can take up to two years to develop a mapping population. Therefore, genetic studies of race structure and pathogenicity require novel approaches and are usually performed on the asexual urediniospore stage of the pathogen's life cycle.

Genomic resources for fungal pathogens provide valuable research tools in understanding the dynamics of plant-pathogen interactions (Dean et al., 2005; Kamper et al., 2006; Cantu et al., 2011; Duplessis et al., 2011a; Fernandez et al., 2012). Hacquard et al. recently published a genome-wide analysis of the poplar leaf rust pathogen (*Melampsora larici-populina*) small secreted proteins, detailing 29 different secreted cysteine repeat-containing protein families with a total of 228 proteins represented (Hacquard et al., 2012). With the availability of *Pt* and *P. graminis* f. sp. *tritici* (wheat stem rust fungus, *Pgt*) reference genomes (http://www.broadinstitute.org/scientific-community/data) a search for the same patterns in the predicted proteomes of these species revealed only four and eleven proteins, respectively. Of these, only two were predicted to be secreted in *Pt* and nine in *Pgt. graminis* (Bruce et al, unpublished data). This indicates that the *Puccinia* group may not use the same effector set as the *Melampsora* group, which may be related to differences between their host plants. To date, only one avirulence effector from cereal rusts has been verified (Nirmala et al., 2011). The presence of two rust proteins from *Pgt* is recognized by the barley resistance protein, RPG1. The rust proteins directly interact with RPG1 in yeast two hybrid experiments and activate an RPG1-mediated hypersensitive response (Nirmala et al., 2011).

With the rapidly decreasing cost of genome and transcriptome sequencing, understanding the mechanisms of pathogenesis and virulence in these organisms is becoming less difficult. In this study, six *Pt* races were inoculated on a susceptible host. Six days after inoculation (DAI), leaves were harvested and RNA extracted. Transcript-enriched RNA was sequenced using Illumina next generation sequencing and the resulting reads assembled. To identify potential fungal effectors, amino acid changes found within secreted peptides were identified in the assembly and correlated to the virulence patterns observed for the races. Using this approach, we have identified 15 candidate avirulence effectors and characterized their expression during the infection process.

## Results

**Figure 1:**
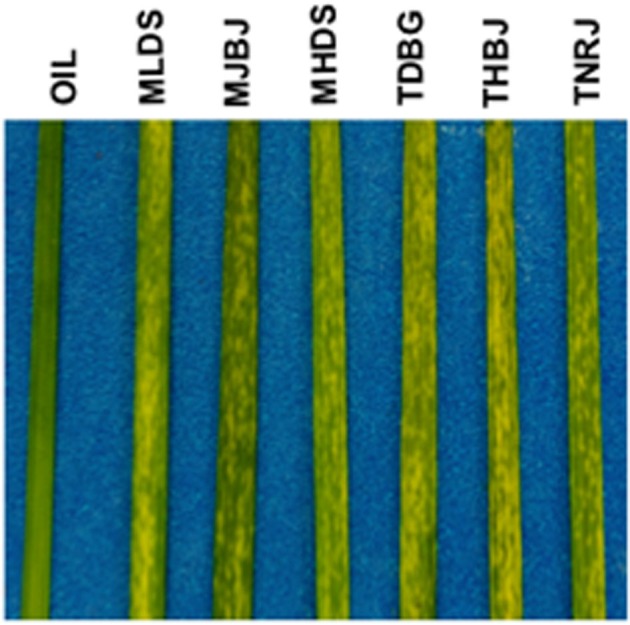
**Infection phenotypes of six *P. triticina* races on the susceptible spring wheat cultivar Thatcher at six days post inoculation, before sporulating pustules appear.** Races are listed at the top. Oil represents the oil only control.

The six *Pt* targeted races were all found in North America and their avirulence/virulence combinations are listed in Table 1. MHDS and MLDS belong to North American lineage 3 (NA3; Tremblay et al., 2013) and were collected in 2004 in Kansas and Ohio, respectively. These two races only differ in their reaction to *Lr 9* and *Lr16* using the standard differential set. MJBJ, THBJ, TDBG, and TNRJ belong to lineage NA5 and were collected in 1997 in Nebraska, 2005 in Texas, 2004 in Texas and 2004 in Kansas, respectively (Tremblay et al., 2013). Each are much more varied in their reactions to the differential wheat lines. TNRJ was the most virulent *Pt* race at the time this study was started. Wheat plants from the susceptible cultivar Thatcher (Tc) were inoculated separately, with each of the six rust races. The inoculations were heavy with a majority of the leaf area showing a significant infection reaction at 6 days post inoculation. Pustule formation was apparent, but urediniospores had not erupted (Figure 1).

**Table 1:**
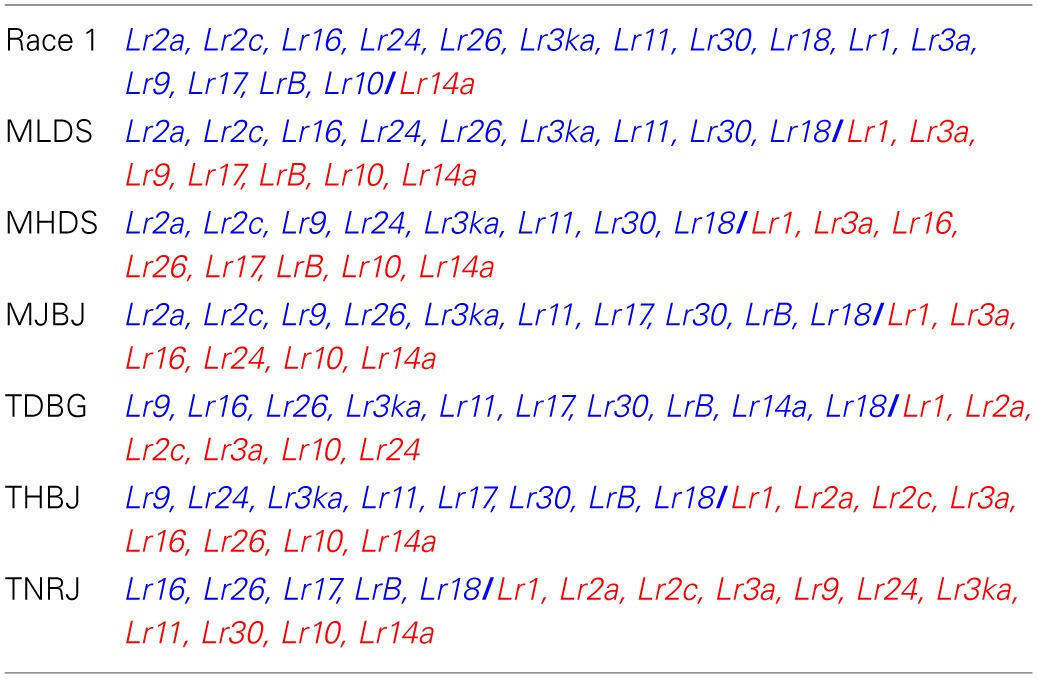
**Listing of *P. triticina* races used in the experiment**.

Races are named based on their reaction to leaf rust resistance gene (Lr) differential Thatcher isolines as described by McIntosh et al. (1995). Lr genes with a low infection type (IT = 0; resistant) are highlighted in blue and Lr genes with a high infection type (IT = 3–4) susceptible are in red. Race1 has the pathotype BBBD.

Following inoculation and incubation, leaves with heavy infections were harvested and RNA was extracted. Total RNA was treated to remove ribosomal RNA, without requiring a polyA selection. Transcriptome RNA representing the wheat and leaf rust transcriptomes was fragmented and used to generate first and second strand cDNA. The second strand cDNA was size-fractionated and amplified in gel prior to Illumina sequencing. A total of 165 million raw reads were generated by Illumina sequencing using standard parameters with paired end 60 bp reads. The sequenced transcriptomes were assembled using Trinity (Grabherr et al., 2011). The transcriptomes were separated into wheat-associated and leaf rust-associated files by aligning the resulting assembled contigs to the TIGR wheat EST database (available at http://www.jcvi.org) or to the leaf rust draft genome V2 of Race1 (pathotype BBBD, http://www.broadinstitute.org/scientific-community/data). A total of 222,571 leaf rust contigs were identified from the assembled contigs. Statistics for the individual races are found in Table 2.

**Table 2:** **Illumina RNAseq data and assembly statistics**.

**Race**	**Raw reads**	**Mb reads**	**Contigs**	**N25 (bp)**	**N50 (bp)**	**N75 (bp)**	**Max (bp)**
MHDS	26419162	3.170 Mb	38192	1492	817	471	12254
MLDS	25556420	3.066 Mb	35568	1719	927	512	10747
MJBJ	23415788	2.809 Mb	34528	1646	890	490	13792
TDBG	27731985	3.327 Mb	36281	1705	929	503	8860
THBJ	33225893	3.987 Mb	39509	1852	1034	555	10136
TNRJ	28404510	3.408 Mb	38673	1454	824	485	10696

**Figure 2:**
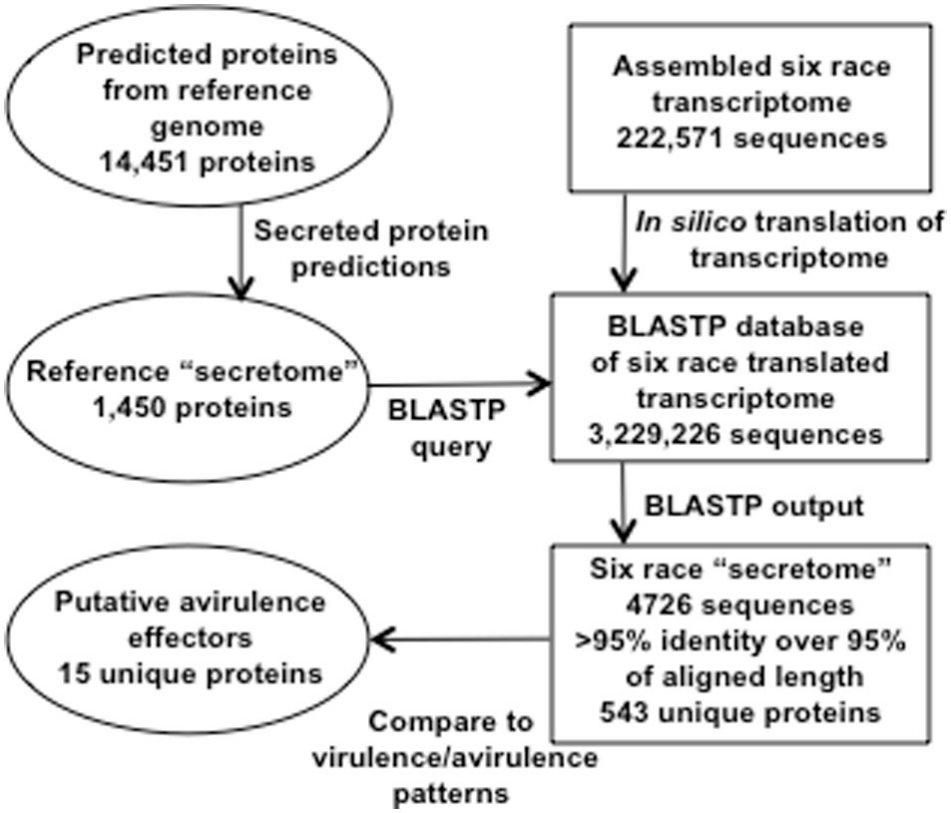
**Schematic of bioinformatic pipeline used to identify 15 secreted peptides that are candidates for avirulence effectors**.

The bioinformatic workflow for identification of secreted proteins in RNA-Seq transcripts is represented in Figure 2. SignalP, TargetP, and TMHMM prediction algorithms were used to determine which proteins among the predicted genes in the race 1 reference genome may be secreted (Petersen et al., 2011). Six frame translations of the assembled transcripts from the six races were produced *in silico* to make a set of amino acid sequences from the assembly data. There were 1450 predicted Race1 (BBBD) secreted amino acid sequences. These were used as a BLASTP query against the six race translated transcriptome (Altschul et al., 1990). The BLAST results were parsed using a custom Python script [http://www.python.org, (Cock et al., 2009)] to extract the Race1 (BBBD) identifier, the six race protein identifier, number of positive matches, number of identity matches and the alignment length. There were a total of 4726 alignments from the six race proteins with an identity/alignment length ratio greater than 0.95, of these, 543 unique secreted proteins were found across the six races, and 445 were shared by all six races (Supplementary Table 1).

To determine whether or not the virulence patterns observed could be correlated to non-synonymous protein changes, the BLASTP query results were parsed and filtered using a Python script [http://www.python.org, (Cock et al., 2009)] to extract gene identifiers, unique race identifiers and match quality. This data was filtered by race to generate lists of gene names that matched the reference sequence exactly, or had at least one SNP resulting in a non-synonymous amino acid substitution. As the reference Race1 (BBBD) is avirulent on 15 out of 16 of the resistance genes in the differential set, proteins that matched the reference exactly were assigned to a list matching races with a low infection type, incompatible interaction. Proteins with SNPs resulting in non-synonymous codon changes were associated with a high infection type compatible interaction. For example, PTTG_05760 had significant matches in all six races. The hits in MLDS, TDBG, and TNRJ matched the Race1 (BBBD) protein sequence exactly, while hits from MHDS, MJBJ, and THBJ had a non-synonymous amino acid substitution (serine to alanine). Race1 (BBBD), MLDS, TDBG, and TNRJ all have a low (avirulent) infection type on TcLr16. MHDS, MJBJ, and THBJ all have a high (virulent) infection type on TcLr16. Thus, the analysis considers this protein to be a candidate avirulence protein. This analysis identified 15 unique secreted peptides (Table 3). Functions for these proteins were predicted using PHYRE2 (Kelley and Sternberg, 2009). Twelve proteins had no significant similarities in the database. One showed significant similarity to cerato-platanin, a fungal elicitor in the Barwin-like endoglucanase superfamily (Pazzagli et al., 1999). A second protein contained a tyrosinase domain and the last showed significant similarity to a gibberellin receptor.

**Table 3:** **Putatively secreted peptides corresponding to virulence shifts**.

**Gene name^a^**	**Putative function^b^**	**Corresponding *Lr* genes^c^**
PTTG_05870	Unknown	*Lr2a, Lr2c, Lr9, Lr3ka, Lr11, Lr30*
PTTG_00023	Unknown	*Lr9, Lr3ka, Lr11, Lr30*
PTTG_25426	Unknown	*Lr9, Lr3ka, Lr11, Lr30*
PTTG_28391	Unknown	*Lr9, Lr3ka, Lr11, Lr30*
PTTG_11899	Unknown	*Lr9, Lr3ka, Lr11, Lr30*
PTTG_03539	Tyrosinase	*Lr9, Lr3ka, Lr11, Lr30*
PTTG_05706	Unknown	*Lr16*
PTTG_12153	Unknown	*Lr16, Lr26*
PTTG_12522	Unknown	*Lr16, Lr26*
PTTG_25271	Barwin-like endoglucanase^d^	*Lr16*
PTTG_26277	Unknown	*Lr16, Lr26, Lr17, LrB*
PTTG_09426	Unknown	*Lr16, Lr26*
PTTG_25509	Gibberellin receptor^e^	*Lr24*
PTTG_25269	Unknown	*Lr17, LrB*
PTTG_02284	Unknown	*Lr16*

a Gene names are from the version 2 annotation of the leaf rust genome (http://www.broadinstitute.org/scientific-community/data).

b Putative functions as predicted by PHYRE2 (Kelley and Sternberg, 2009). Functions with a partial coverage confidence of 100% are reported.

c Leaf rust resistance genes matching the virulence/avirulence pattern for the protein presented as determined by the presence of non-synonymous amino acid substitutions among the six races.

d Barwin-like endoglucanase protein superfamily includes the fungal elicitor cerato-platanin family (Pazzagli et al., 1999).

e Partial coverage of the amino acid sequence (22%) was predicted to have structure similarity to a probable gibberellin receptor. Other high confidence hits included hormone-sensitive esterases and lipases.

**Figure 3:**
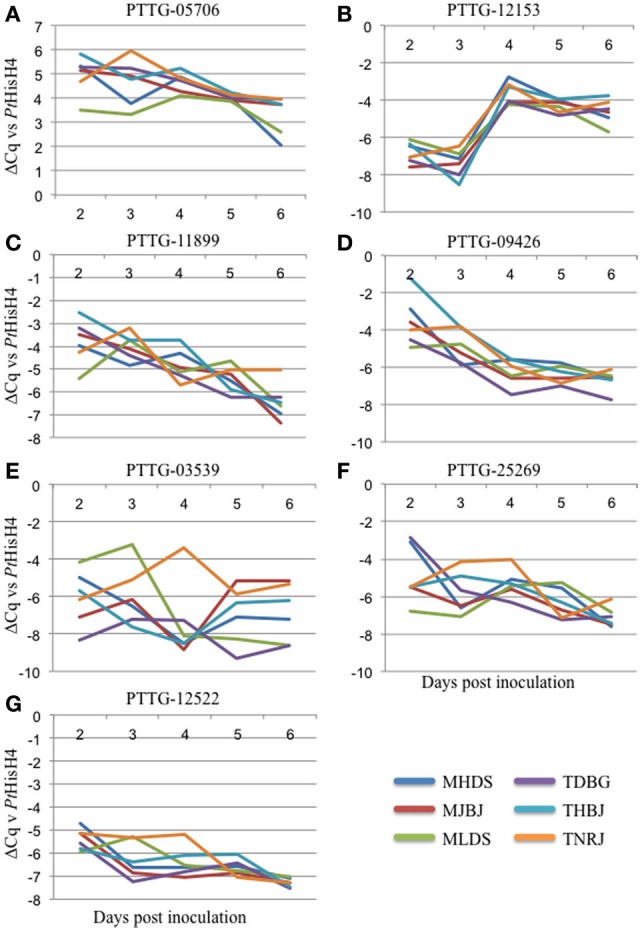
**Gene expression profiles for seven avirulence candidates from *P. triticina*.** Plant samples were taken at 24 h intervals post inoculation (X axis). Real-time PCR was used to quantify expression of each gene. *P. triticina* Histone H4 (PtHisH4) was used as the internal control for normalization. ΔCq vs. PtHisH4 was used to plot expression (Y axis).

qRT-PCR primers were designed for the 15 genes identified as putative avirulence effectors. These were tested on cDNA generated from RNA from Thatcher wheat inoculated separately with the six leaf rust races and collected daily for 6 days. Following testing for specificity and appropriate efficiency, the primers were used to measure expression of seven genes. ΔCq was calculated against the rust reference gene histone H4 (PtHisH4). PtHisH4 was chosen for its stable expression among the races. Temporal expression profiles for these genes are presented in Figures 3A–G. There were various expression patterns with the seven genes, but were similar within all six races. PTTG_05706 was transcribed at a higher rate than PtHisH4 and expression was maintained throughout the 6 days (Figure 3A). PTTG_12153 was expressed at a low level for the first 3 days, but transcription spiked at day 4 and maintained this level through day 6. PTTG_11899 and PTTG_09426 both showed a general trend of reduced transcription from day 2 to day 6 (Figures 3C,D). The final three genes had differences in expression between the races. MLDS had a higher expression of PTTG_03539 at day 3, while TNRJ spiked at day 4 while the other five races had very low expression. Expression of PTTG_03539 varied between all races at day 5 and 6 (Figure 3E). TDBG and MHDS expressed PTTG_25269 at a high rate on day 2, while TNRJ was higher at day 3 (Figure 3F). The peak expression of PTTG_12522 was steady for TNRJ till day four (Figure 3G).

## Discussion and future directions

Secreted peptides of pathogenic fungi are of particular interest as some have been demonstrated to act as avirulence effectors in other studied systems (Dodds et al., 2004; De Wit et al., 2009; Nirmala et al., 2011). In order to advance knowledge of the mechanisms underlying virulence and avirulence in the wheat-*Pt* pathosystem, we have employed an RNA-Seq approach to identify genes and proteins that are expressed by *Pt* during compatible interactions and may in turn be perceived by the host during incompatible interactions. To date, there are 88 wheat leaf rust resistance genes described and used in various breeding programs (http://www.shigen.nig.ac.jp/wheat/komugi/, accessed 30 September 2013). Of these, at least 27 have been backcrossed into the Thatcher background, providing a uniform genetic background against which their contribution to disease resistance can be assessed (Kolmer, 1996). Because of the biotrophic nature of the fungus, these isolines are also the only means of studying the differing virulence reactions to resistance genes. Sixteen of these isolines are routinely used as differentials for race determination during annual surveys. The races' differential reaction to the presence of these resistance genes will allow for identification of potential avirulence effectors represented in the transcriptomes.

RNA sequencing has been employed to identify *Pt* proteins involved during infection. At 6 days post inoculation, many of the genes needed for infection are presumed to be active. Urediniospores are being formed, feeding structures are established, but hyphae are growing and haustoria are still being formed. Isolating RNA at this stage would presumably represent many of the genes of the asexual cycle. This study was focused on identifying the secretome of *Pt* and variants associated with virulence shifts. We identified 543 expressed genes that may code for secreted peptides. The six races in this study shared 445 of those genes. The remaining 98 genes, while not represented in the assembly among all six races, may still be expressed in individual races. For example, while contigs matching PTTG_25269 were only found in three of the six races, the qRT-PCR data indicated that the gene representing this contig was expressed in all of the races. This is an important point to consider when analyzing transcriptome assemblies; the representation of the transcriptome may not be complete, depending on the sequencing depth.

Among 543 expressed genes, SNPs resulting in non-synonymous amino acid changes relative to the reference race 1 predicted proteome were observed in the assembly data that correlated with virulence shifts for 11 different leaf rust resistance genes. Since the reference Race1 (BBBD) is avirulent on 15 of the 16 leaf rust genes tested, the virulence shift predictions were based on the avirulent races having the reference race-type allele, and the virulent races having an alternate allele that may evade recognition by the resistance gene, or have an unrecognized altered activity.

Of the 15 identified, three had high confidence functional predictions from PHYRE2 (Kelley and Sternberg, 2009), including a tyrosinase, a putative gibberellin receptor, and a Barwin-like endoglucanase. Appropriately performing qRT-PCR primers were developed for seven of the genes. The expression profiles for these genes showed differences that may correlate with their activity. For example, PTTG_12153 is upregulated between three and four DAI. This corresponds to a shift from mainly haustorial feeding to urediniospore production. While the function of this gene is currently unknown, future studies may indicate a role in this capacity. One of the proteins has structural similarity to a Barwin-like endoglucanase, a family containing cerato-platanin, a known fungal elicitor (Pazzagli et al., 1999). In *Arabidopsis*, ectopic expression of this elicitor has protective effects against other pathogens (Yang et al., 2009). However, we were unable to develop qRT-PCR primers to detect expression for this gene. In the future, it will be cloned and characterized from genomic DNA. PTTG_05706 showed the highest level of expression, was rapidly induced within the first 48 h, and maintained a high expression level throughout the course of infection. PTTG_12522 was induced at two DAI, and showed stable expression for the remainder of the measured period. Since wheat leaf rust completes its entire infection cycle in the 6 day timeframe (penetration, haustoria formation, uredinia production, pre-pustule eruption), the data presented here should be a good indication of when and how these genes are functioning during infection. PTTG_03539 shows a spike in expression for race TNRJ at day 4 relative to the other races tested. Additionally, PTTG_25269 shows a spike and sustained high expression in TNRJ in days 3 and 4. The altered expression of these genes in this race may be responsible for its observed aggressive nature in the field. Five random leaves were selected for RNA extraction at each time point. This sampling scheme normalizes plant-to-plant variation in the expression data. Genes showing interesting changes in expression patterns, such as PTTG_03539, will be examined more closely in the future. Temporally variable expression of secreted proteins that may be effectors or avirulence genes of rust pathogens in different plant species has been observed in other studies, suggesting that these genes may affect host colonization or recognition of the pathogen by the host in a time-dependent manner (Duplessis et al., 2011b; Cantu et al., 2013; Tremblay et al., 2013).

The work reported here generated six secretomes of *Pt* including 543 predicted secreted proteins. RNAseq allows a much deeper sampling of the RNA species and resulting in a higher probability of an RNA to be identified. However, our work shows that assemblies may not reliably detect low level transcripts. Association of SNPs with virulence shifts have shown that 11 of the 15 avirulence candidates could correlate to reactions by multiple resistance genes. More races from known genetic lineages need to be sequenced so that stronger associations can be made. The putative effector genes in this study will be cloned from cDNA and used in experiments to determine if they have a role in the resistance response in wheat. A better understanding of the molecular determinants in disease resistance and susceptibility can generate additional tools for practical application in breeding programs and race identification.

## Materials and methods

### Plant growth conditions, inoculation, and tissue harvest

The hard red spring wheat variety, Thatcher (*Triticum aestivum* L.) was used in all of the experiments. Seeds were planted in Metro Mix 360 (SunGro, Vancouver, Canada) and grown in a growth chamber at 18°C with 16 h day/8 h night cycles. At the 2–3 leaf stage, plants were inoculated by suspending 5 mg urediniospores per mL Soltrol 170 (Philips 66, Bartlesville, OK) and spraying onto the plants using an atomizer at 40 psi. Following inoculation, plants were incubated in a dew chamber at 100% humidity for 24 h at 18°C. Plants were then moved back into growth chambers. Leaves from 30 inoculated plants per race were collected and pooled 6 DAI and immediately frozen in liquid nitrogen. For time course expression studies, 30 plants were inoculated per race as described. Five random leaves per race were collected and stored as described at 2, 3, 4, 5, and 6 DAI. Total RNA was extracted from tissue with the *mir*Vana miRNA isolation kit (AM1560, Life Technologies, Carlsbad, CA) according to the manufacturer's recommendations with the omission of the miRNA enrichment step. RNA was quantified with a Nanodrop ND1000 spectrophotometer (Thermo Fisher Scientific, Waltham, MA).

### RNA sequencing

Total RNA was submitted to Cofactor Genomics (St. Louis, MO). The RNA was sequenced according to in house protocols steps summarized as follows. Whole transcriptome RNA was extracted from total RNA by removing large and small ribosomal subunit RNA (rRNA) using the RiboMinus Eukaryote Kit (Invitrogen, Carlsbad, CA). 5 μg of total RNA was hybridized to rRNA-specific biotin labeled probes at 70°C for 5 min. The rRNA-probe complexes were removed by strepavidin-coated magnetic beads. The rRNA-free transcriptome RNA was concentrated by ethanol precipitation. Double-stranded cDNA was treated with a mix of T4 DNA polymerase, Klenow large fragment and T4 polynucleotide kinase to create blunt-ended DNA, to which was subsequently added a single A base at the 3′ end using Klenow fragment (3′ to 5′ exo-) and dATP. A-tailed DNA was ligated with paired end adaptors using T4-DNA ligase provided with the Illumina RNA-seq kit (Illumina, San Diego, CA). Size selection of adaptor-ligated cDNA was performed by cutting the target fragment out of a 4–12% acrylamide gel. The amplified cDNA library with ideal fragment size was obtained by in-gel PCR using the Phusion High-Fidelity system (New England Biolabs, Ipswitch, MA). Size-fractionated, amplified DNA was sequenced according to the Illumina RNAseq protocol (Illumina).

### cDNA synthesis—illumina sequencing

1 μg of rRNA depleted RNA was fragmented by incubation in fragmentation buffer included in the Illumina RNA-seq kit (Illumina) for 5 min at 94°C. Fragmented RNA was purified by ethanol precipitation. First strand cDNA was prepared by priming the fragmented RNA with random hexamers, followed by reverse transcription with Superscript II (Invitrogen, Carlsbad, CA). Second strand cDNA was synthesized by incubating first strand cDNA with second strand buffer, RNase Out and dNTP from the Illumina RNA-seq kit on ice for 5 min. The reaction mix was then treated with DNA Pol I and RNaseH at 16°C for 2.5 h (Invitrogen).

### Data processing and assembly

Bases with quality scores less than 20 were trimmed from both ends of raw sequencing reads (fastq_quality_trim -q 20 -t 30). Trimmed reads with a length greater than 30 and 80% of bases with quality scores greater than 20 were retained for assembly (fastq_quality_filter -p 20 -q 80). These tools are part of the FASTX-Toolkit (http://hannonlab.cshl.edu/fastx_toolkit/). Quality-filtered reads were assembled into transcripts using Trinity v2011059 (Grabherr et al., 2011), a de Brujin graph-based assembler. The -jaccard_clip option was used to minimize fusion transcripts resulting from overlapping UTR regions from the fungal transcriptome. Assembled transcripts were aligned against the *Pt* Race1 (BBBD) reference genome and retained in a separate FASTA file.

Six frame translations of the six race transcriptome was conducted with a custom Python script [http://www.python.org, (Cock et al., 2009)]. Peptides greater than 20 amino acids in any frame were written to a text file and converted to a BLASTP (Altschul et al., 1990) database. Predicted proteins from the race 1 reference genome were used to generate a list of putatively secreted proteins using SignalP (Petersen et al., 2011). Proteins meeting criteria defined by Joly et al. (2010) as putatively secreted were used in a BLASTP query (Altschul et al., 1990) against the six race translated transcriptome using an expect value of 1e-30. A Python script (http://www.python.org) was used to parse the resulting BLASTP XML output. To identify proteins with SNPs relative to the race 1 reference, a ratio of identical matches over the aligned length was taken from the parsed BLASTP output. Proteins in any race with a ratio less than one were chosen for further analysis.

### Expression profiling, cDNA synthesis—qRT-PCR

First strand cDNA was prepared by priming 1 μg total RNA with random hexamers, followed by reverse transcription with Superscript II (Invitrogen) according to the manufacturer's recommendations. Primers for qRT-PCR were designed from the assembled contigs and used to assess differences in gene expression between the races. Bullseye EvaGreen qPCR mastermix for iCycler (BioRad, La Jolla, CA) was used in all reactions. Three technical replicates were performed for each reaction. All primers were assayed for efficiency prior to use in experiments and had efficiencies within the range 90–110%. The resulting Cq value for the target gene was subtracted from the Cq value of the internal rust reference gene histone H4. Primer sequences used in this study are provided in Supplementary Table 2.

Mention of a trademark of a proprietary product does not constitute a guarantee of warranty of the product by the United States Department of Agriculture, and does not imply its approval to the exclusion of other products that may also be suitable. USDA is an equal opportunity provider and employer.

## Conflict of interest statement

The authors declare that the research was conducted in the absence of any commercial or financial relationships that could be construed as a potential conflict of interest.

## Supplementary material

Supplementary Table 1 **Putatively secreted proteins shared by six races**.

Supplementary Table 2 **Primers used in this study**.
