# Supplementary material for: Using transcription of six Puccinia triticina races to identify the effective secretome during infection of wheat
Source: Front Plant Sci. 2014 Jan 13;4:520. doi: 10.3389/fpls.2013.00520 (PMC3888938; doi:10.3389/fpls.2013.00520)
Supplement: Supplementary Table 1 — Putatively secreted proteins shared by six races. [file DataSheet1.PDF]

Supplementary Table 1 - Putatively secreted proteins shared by six races

	PTTG_01334T0	PTTG_03549T0	PTTG_05589T0
PTTG_00016T0	PTTG_01340T0	PTTG_03570T0	PTTG_05697T0
PTTG_00018T0	PTTG_01364T0	PTTG_03623T0	PTTG_05707T0
PTTG_00056T0	PTTG_01476T0	PTTG_03653T0	PTTG_05715T0
PTTG_00074T0	PTTG_01527T0	PTTG_03747T0	PTTG_05750T0
PTTG_00106T0	PTTG_01578T0	PTTG_03809T0	PTTG_05757T0
PTTG_00108T0	PTTG_01715T0	PTTG_03867T0	PTTG_05844T0
PTTG_00108T1	PTTG_01781T0	PTTG_03917T0	PTTG_05911T0
PTTG_00114T0	PTTG_01803T0	PTTG_03978T0	PTTG_05912T0
PTTG_00127T0	PTTG_01805T0	PTTG_04059T0	PTTG_05946T0
PTTG_00140T0	PTTG_01821T0	PTTG_04155T0	PTTG_05954T0
PTTG_00184T0	PTTG_01827T0	PTTG_04158T0	PTTG_05975T1
PTTG_00286T0	PTTG_01892T0	PTTG_04226T0	PTTG_06020T0
PTTG_00365T0	PTTG_01892T1	PTTG_04227T0	PTTG_06072T0
PTTG_00386T0	PTTG_01990T0	PTTG_04247T0	PTTG_06087T0
PTTG_00398T0	PTTG_01995T0	PTTG_04297T0	PTTG_06113T0
PTTG_00399T0	PTTG_02013T0	PTTG_04308T0	PTTG_06143T0
PTTG_00452T0	PTTG_02051T0	PTTG_04313T0	PTTG_06164T0
PTTG_00455T0	PTTG_02061T0	PTTG_04346T0	PTTG_06237T0
PTTG_00459T0	PTTG_02095T0	PTTG_04406T0	PTTG_06270T0
PTTG_00495T0	PTTG_02125T0	PTTG_04542T0	PTTG_06314T0
PTTG_00528T0	PTTG_02175T0	PTTG_04560T0	PTTG_06324T0
PTTG_00664T0	PTTG_02176T0	PTTG_04590T0	PTTG_06334T0
PTTG_00671T0	PTTG_02186T0	PTTG_04631T0	PTTG_06379T0
PTTG_00675T0	PTTG_02209T0	PTTG_04631T1	PTTG_06434T0
PTTG_00700T0	PTTG_02371T0	PTTG_04631T2	PTTG_06505T0
PTTG_00841T0	PTTG_02372T0	PTTG_04666T0	PTTG_06512T0
PTTG_00846T0	PTTG_02398T0	PTTG_04698T0	PTTG_06571T0
PTTG_00907T0	PTTG_02450T0	PTTG_04843T0	PTTG_06591T0
PTTG_00930T0	PTTG_02461T0	PTTG_04880T0	PTTG_06619T0
PTTG_00931T0	PTTG_02492T0	PTTG_04892T0	PTTG_06722T0
PTTG_00943T0	PTTG_02745T0	PTTG_04985T0	PTTG_06727T0
PTTG_00946T0	PTTG_02758T0	PTTG_05048T0	PTTG_06852T0
PTTG_00946T1	PTTG_02795T0	PTTG_05086T0	PTTG_06910T0
PTTG_00973T0	PTTG_02883T0	PTTG_05116T0	PTTG_06928T0
PTTG_00977T0	PTTG_02917T0	PTTG_05202T0	PTTG_06943T0
PTTG_01002T1	PTTG_02944T0	PTTG_05228T0	PTTG_07032T0
PTTG_01019T0	PTTG_02944T1	PTTG_05378T0	PTTG_07039T0
PTTG_01024T0	PTTG_03033T0	PTTG_05423T0	PTTG_07082T0
PTTG_01032T0	PTTG_03168T0	PTTG_05432T0	PTTG_07231T0
PTTG_01057T0	PTTG_03287T0	PTTG_05432T1	PTTG_07281T0
PTTG_01125T0	PTTG_03308T0	PTTG_05438T0	PTTG_07281T1
PTTG_01156T0	PTTG_03381T0	PTTG_05451T0	PTTG_07345T0
PTTG_01202T0	PTTG_03497T0	PTTG_05518T0	PTTG_07352T0
PTTG_01303T0	PTTG_03497T1	PTTG_05544T0	PTTG_07365T0

PTTG_07376T0	PTTG_10000T0	PTTG_12393T0	PTTG_26567T0
PTTG_07398T0	PTTG_10097T0	PTTG_12408T0	PTTG_26662T0
PTTG_07571T0	PTTG_10178T0	PTTG_12430T0	PTTG_26786T0
PTTG_07591T0	PTTG_11645T0	PTTG_12445T0	PTTG_26817T0
PTTG_07705T0	PTTG_11646T0	PTTG_12483T0	PTTG_26825T0
PTTG_07795T0	PTTG_11654T0	PTTG_12486T0	PTTG_26831T0
PTTG_07799T0	PTTG_11656T0	PTTG_12499T0	PTTG_26916T0
PTTG_07819T0	PTTG_11671T0	PTTG_12514T0	PTTG_26994T0
PTTG_07819T1	PTTG_11686T0	PTTG_12533T0	PTTG_27145T0
PTTG_07885T0	PTTG_11690T0	PTTG_12541T0	PTTG_27172T0
PTTG_07972T0	PTTG_11693T0	PTTG_12542T0	PTTG_27231T0
PTTG_08011T0	PTTG_11707T0	PTTG_12601T0	PTTG_27277T0
PTTG_08198T0	PTTG_11740T0	PTTG_12649T0	PTTG_27303T0
PTTG_08256T1	PTTG_11770T0	PTTG_12651T0	PTTG_27311T0
PTTG_08279T0	PTTG_11847T0	PTTG_12693T0	PTTG_27350T0
PTTG_08315T0	PTTG_11858T0	PTTG_12693T1	PTTG_27401T0
PTTG_08315T1	PTTG_11858T1	PTTG_12701T0	PTTG_27417T0
PTTG_08334T0	PTTG_11922T0	PTTG_12724T0	PTTG_27471T0
PTTG_08343T0	PTTG_11960T0	PTTG_12725T0	PTTG_27510T0
PTTG_08363T0	PTTG_11963T0	PTTG_12726T0	PTTG_27510T1
PTTG_08453T0	PTTG_11990T0	PTTG_12758T0	PTTG_27521T0
PTTG_08454T0	PTTG_11995T0	PTTG_25130T0	PTTG_27545T0
PTTG_08460T0	PTTG_12042T0	PTTG_25166T0	PTTG_27668T0
PTTG_08468T0	PTTG_12051T0	PTTG_25228T0	PTTG_27679T0
PTTG_08503T0	PTTG_12066T0	PTTG_25247T0	PTTG_27688T0
PTTG_08505T0	PTTG_12096T0	PTTG_25256T0	PTTG_27691T0
PTTG_08509T0	PTTG_12125T0	PTTG_25262T0	PTTG_27704T0
PTTG_08539T0	PTTG_12133T0	PTTG_25295T0	PTTG_27769T0
PTTG_08542T0	PTTG_12137T0	PTTG_25377T0	PTTG_27808T0
PTTG_08580T0	PTTG_12156T0	PTTG_25454T0	PTTG_27828T0
PTTG_08596T0	PTTG_12170T0	PTTG_25496T0	PTTG_27844T0
PTTG_08890T0	PTTG_12171T1	PTTG_25766T0	PTTG_28035T0
PTTG_08890T1	PTTG_12184T0	PTTG_25922T0	PTTG_28070T0
PTTG_08963T0	PTTG_12189T0	PTTG_25982T0	PTTG_28256T0
PTTG_09007T0	PTTG_12201T0	PTTG_26038T0	PTTG_28265T0
PTTG_09055T0	PTTG_12208T0	PTTG_26187T0	PTTG_28296T0
PTTG_09087T0	PTTG_12223T0	PTTG_26282T0	PTTG_28305T0
PTTG_09171T0	PTTG_12230T0	PTTG_26290T0	PTTG_28341T0
PTTG_09239T0	PTTG_12299T0	PTTG_26296T0	PTTG_28361T0
PTTG_09239T1	PTTG_12299T1	PTTG_26353T0	PTTG_28380T0
PTTG_09281T0	PTTG_12333T0	PTTG_26395T0	PTTG_28386T0
PTTG_09414T0	PTTG_12333T1	PTTG_26483T0	PTTG_28442T0
PTTG_09501T0	PTTG_12337T0	PTTG_26503T0	PTTG_28461T0
PTTG_09548T0	PTTG_12337T1	PTTG_26516T0	PTTG_28488T0
PTTG_09712T0	PTTG_12354T0	PTTG_26533T0	PTTG_28654T0
PTTG_09930T0	PTTG_12380T0	PTTG_26540T0	PTTG_28659T0

PTTG_28663T0	PTTG_00662T0
PTTG_28700T0	PTTG_25253T0
PTTG_28915T0	PTTG_02264T0
PTTG_29065T0	PTTG_06256T0
PTTG_29075T0	PTTG_12505T0
PTTG_29082T0	PTTG_03568T0
PTTG_29272T0	PTTG_05428T0
PTTG_29361T0	PTTG_02155T0
PTTG_29432T0	PTTG_27630T0
PTTG_29669T0	PTTG_00522T0
PTTG_29749T0	PTTG_25160T0
PTTG_30061T0	PTTG_26127T0
PTTG_30109T0	PTTG_12400T0
PTTG_30323T0	PTTG_05992T0
PTTG_30323T1	PTTG_11739T0
PTTG_30753T0	PTTG_00174T0
PTTG_02663T0	PTTG_00015T0
PTTG_02663T1	PTTG_01189T0
PTTG_04458T0	PTTG_11655T0
PTTG_01431T0	PTTG_05189T0
PTTG_28904T0	PTTG_04474T0
PTTG_28602T0	PTTG_11699T0
PTTG_25762T0	PTTG_26482T0
PTTG_27607T1	PTTG_02919T0
PTTG_26977T0	PTTG_29866T0
PTTG_04362T0	PTTG_06895T0
PTTG_05735T0	PTTG_00617T0
PTTG_09620T0	PTTG_25968T0
PTTG_00666T0	PTTG_28044T0
PTTG_04222T0	PTTG_25643T0
PTTG_06474T0	PTTG_10998T0
PTTG_08285T0	PTTG_10691T0
PTTG_03567T0	PTTG_05699T0
PTTG_01370T0	PTTG_05907T0
PTTG_12075T0	PTTG_11512T0
PTTG_30007T0	PTTG_07195T0
PTTG_03093T0	
PTTG_07673T0	
PTTG_28156T0	
PTTG_06573T0	
PTTG_06325T0	
PTTG_03866T0	
PTTG_09831T0	
PTTG_08735T0	
PTTG_10273T0	
PTTG_01186T0	
