# Supplementary material for: Using transcription of six Puccinia triticina races to identify the effective secretome during infection of wheat
Source: Front Plant Sci. 2014 Jan 13;4:520. doi: 10.3389/fpls.2013.00520 (PMC3888938; doi:10.3389/fpls.2013.00520)
Supplement: Supplementary Table 2 — Primers used in this study. [file DataSheet2.PDF]

Supplementary Table 2 - Primers used in this study

Primer	Sequence
Pt_HisH4-1-qF	GGATCTCCGGTCTGATCTAT
Pt_His_H4-1-qR	TTGGCATGTTCAGTGTAAGT
PTTG_05870-1qF	AGTTCTGGGAGGAGATAAAG
PTTG_05870-1qR	ATCCTTGTTGATCTGAAAGAC
PTTG_11899-1qF	CTGCGACGAATATAGCAAG
PTTG_11899-1qR	TTTGCATCGACATCACTATC
PTTG_03539-1qF	CGGTCCATCGAATGTATTG
PTTG_03539-1qR	GGAAGTTTCCAGCATCAC
PTTG_05706-1qF	CCAAGGGTCATACTACCATA
PTTG_05706-1qR	TTAGGTTCAGCCTCCTTC
PTTG_12153-1qF	GTACCGGACACAGGAATA
PTTG_12153-1qR	CCTGTTCTGACTGGAATTG
PTTG_12522-1qF	AGCTCCAAGAAGTGGATAG
PTTG_12522-1qR	TGTGTTTGTAGGGCTAGAT
PTTG_09426-1qF	CTTTCTATCACAGGCGTTTC
PTTG_09426-1qR	GCTTCAGTGGTCAATTCTAATA
PTTG_25269-1qF	GATTGGTACTGAGTTGATTGT
PTTG_25269-1qR	CAATGCTCCCTTGTCTTG
